# Approaches to investigate tissue-resident innate lymphocytes metabolism at the single-cell level

**DOI:** 10.1038/s41467-024-54516-3

**Published:** 2024-11-30

**Authors:** Carrie Corkish, Cristhiane Favero Aguiar, David K. Finlay

**Affiliations:** 1 School of Biochemistry and Immunology, Trinity Biomedical Sciences Institute, Trinity College Dublin, Dublin, Ireland; 2 School of Pharmacy and Pharmaceutical Sciences, Trinity Biomedical Sciences Institute, Trinity College Dublin, Dublin, Ireland

**Keywords:** Innate immune cells, Metabolomics, Systems analysis, Gene regulation in immune cells

## Abstract

Tissue-resident innate immune cells have important functions in both homeostasis and pathological states. Despite advances in the field, analyzing the metabolism of tissue-resident innate lymphocytes is still challenging. The small number of tissue-resident innate lymphocytes such as ILC, NK, iNKT and γδ T cells poses additional obstacles in their metabolic studies. In this review, we summarize the current understanding of innate lymphocyte metabolism and discuss potential pitfalls associated with the current methodology relying predominantly on in vitro cultured cells or bulk-level comparison. Meanwhile, we also summarize and advocate for the development and adoption of single-cell metabolic assays to accurately profile the metabolism of tissue-resident immune cells directly ex vivo.

## Abstract (web summary)

Immune cells pursue metabolic adaptations to accommodate the dynamic changes of their functions. Here in this review the authors summarize immune metabolic states of various innate lymphoid cells and advocate single-cell level methods for studying the complex networks of immune metabolism.

## Introduction

Innate lymphocytes, such as ILCs, NK cells, iNKT cells and γδ T cells, play pivotal roles in tissue homeostasis and immune responses, with each having an affinity for different tissue locations. Understanding their unique metabolic profiles is essential for elucidating their functions and potential therapeutic applications. This review explores the current understanding of the metabolism of tissue-resident innate lymphocytes, comprehensively examines current methodologies, and promotes the utilisation of single-cell metabolic assays directly ex vivo to capture the in vivo metabolic landscape. As little is known about the metabolism of tissue-resident lymphocytes in humans, and because some of the new cutting-edge techniques were developed in mice, this review will primarily focus on our understanding of murine innate lymphocytes. Further, it is worth noting that there are species-specific differences in immune cell metabolism, however, that are yet to be characterised for tissue-resident innate lymphocytes^1,2^.

### Innate lymphocytes in the tissues

**Fig. 1:**
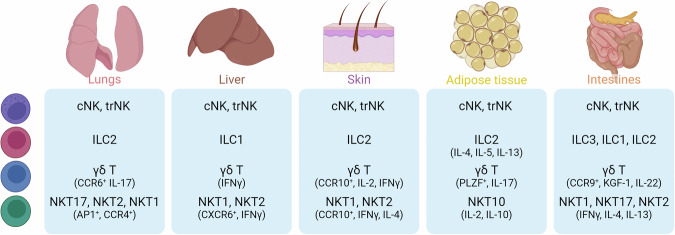
Tissue-resident innate lymphocytes. Distribution of NK, iNKT, ILC and γδ T cells in the lungs, liver, skin, adipose tissue and intestine. For each tissue, the subsets described to be populated by these cells and their specific markers/cytokines are listed based on murine studies. This figure was created in BioRender. Finlay (2024) https://BioRender.com/e69o761.

*Innate lymphoid cells (ILC)* comprise a heterogeneous family of tissue-resident immune cells, with functions in the defence against invading pathogens and transformed cells^3^. Within tissues, ILCs have critical functions in maintaining tissue balance and responding to local inflammation and injuries^4,5^. ILCs are found throughout the body and can be classified into three main groups based on their surface marker expression, cytokine secretion profiles, and transcription factor expression^3,6^ (Table 1 and Fig. 1). Group 1 ILCs, which include natural killer (NK) cells and ILC1s, can be identified through their co-expression of the surface markers NK1.1 and NKp46, the transcription factor T-bet, and the cytokine IFNγ. Further, NK cells and ILC1s can be distinguished by the expression of two transcription factors, EOMES and HOBIT, in NK and ILC1 cells, respectively; similarly, the presence of surface markers CD49b and CD200R1 also helps define NK and ILC1, respectively^3,6–8^. Generally, ILC1s are long-term tissue-resident cells, while NK cells recirculate through the vasculature, performing host surveillance against infections and tumours. However, there is a recent appreciation that tissue-resident NK cells exist, and they have different gene expression patterns from ILC1s^9^. Moreover, there is evidence that upon infection, circulating NK cells can establish residency within the infected tissue, with these tissue-resident NK cells having roles in maintaining tissue homeostasis^10,11^. Group 2 ILCs can be identified through their expression of the transcription factor GATA-3, and through their production of type 2 cytokines such as IL-4, IL-5 and IL-13. Group 2 ILCs contribute to the defence against helminth infection, and have been shown to be involved in the allergic response within the lungs^3,12,13^. Group 3 ILCs, which include ILC3s and lymphoid tissue-inducer (LTi) cells, express RORγt and produce IL-17A and IL-22, with ILC3s providing antibacterial immunity in the intestine, while LTi cells participate in the formation of secondary lymphoid structures during embryonic development^3,5,14^.

**Table 1:** Overview of innate lymphocytes

Immune cell subsets	Nomenclature	Surface markers	Transcription factors	Development	Tissue locations	Cytokine and effector profile
ILC	cNK, trNK	NK1.1, NKp46, CD49b	EOMES, T-bet	Bone marrow	Lymphoid and non-lymphoid tissues	IFNγ, TNFα, cytotoxic molecules
	ILC1	NK1.1, NKp46, CD49a, CD200R1	HOBIT, T-bet	Bone marrow	Non-lymphoid tissues	IFNγ, TNFα
	ILC2	ICOS, IL17Rβ	GATA-3, RORα	Bone marrow	Most abundant in the lung	IL-4, IL-5, IL-9, IL-13, AREG
	ILC3	NKp46, AhR	RORγt, T-bet,	Bone marrow	Most abundant in the gut	IL-17, IL-22, GM-CSF
	LTi	CCR6	RORγt	Bone marrow	Lymphoid tissues	IL-17, IL-22
γδ T cells	γδ^17^, γδ^IFN^	CD27, CD45RB, CD44	RORγt, T-bet,	Thymus	Most abundant in the intestine, skin, brain, adipose tissue	IL-17, IFNγ
iNKT cells	NKT1	CD24^lo^, NK1.1^hi^, CD27^hi^, CXCR3^+^	T-bet	Thymus	Most abundant in liver, spleen, adipose tissue	IFNγ, IL-2, IL-4, IL-10, IL-17, TNF
	NKT2	CD24^lo^, CD27^hi^, CD4^+^	GATA-3, PLZF			
	NKT17	CD24^lo^, CCR6^+^, CD103^hi^, SDC-1^+^	RORγt			
	NKT10	PD-1, ICOS, Nrp1	E4BP4

*γδ T lymphocytes* have features of both innate and adaptive lymphocytes, and account for 0.5–5% of all T cells. γδ T cells encompass various subgroups based on their T cell receptor (TCR) gene usage and cellular function. Alternatively, γδ T cells may also be divided into two groups based on cytokine secretion profiles: CD27^−^IL-17-producing cells (γδ^17^) and CD27^+^IFNy-producing cells (γδ^IFN^) (Table 1). Significant populations of these cells can be found in skin, intestine, brain and adipose tissue^15^ (Fig. 1). In the context of cancer, it is generally thought that γδ^IFN^ cells play roles in tumour surveillance and regression, while γδ^17^ cells promote tumour growth and metastasis^16,17^. The in-depth breakdown of γδ T cell subgroups has been reviewed extensively elsewhere^15,18^.

*Natural killer T (NKT) cells* are a population of unconventional αβ T lymphocytes that recognises lipids presented by the MHC-I-like molecule, CD1d. NKT cells can be subdivided in type I (or invariant NKT cells (iNKT)) and type II NKT cells. While iNKT cells have a semi-invariant TCR, type II NKT cells utilise a more diverse TCR repertoire and are less explored due to the difficulty in identifying them by specific markers^19,20^. Thus, for the purpose of this review, only work on iNKT cells will be discussed. iNKT cells are primarily found in the liver, spleen and adipose tissue, with lesser populations within the lungs, intestines and lymph nodes^21^ (Fig. 1). iNKT cells have been shown to play important roles in controlling infection and tumour development but, due to their tissue-resident nature, are also implicated in maintaining tissue homeostasis^22–25^. iNKT cells can display properties of NK cells, with some subsets expressing the conventional NK cell markers NK1.1 and NKG2D. While conventional T cells are activated by peptides, iNKT cells are activated by glycolipids and are characterised by the rapid production of IFNγ and IL-4. Moreover, iNKT cells have the capacity to produce a broad range of cytokines, including IL-2, IL-10, IL-13, IL-17 and TNF, and chemokines, including RANTES, MIP-1α and MIP-1β (Table 1). In-depth characterisation of the development and function of iNKT cells can be found here^22^.

### Metabolism of innate lymphocytes

**Fig. 2:**
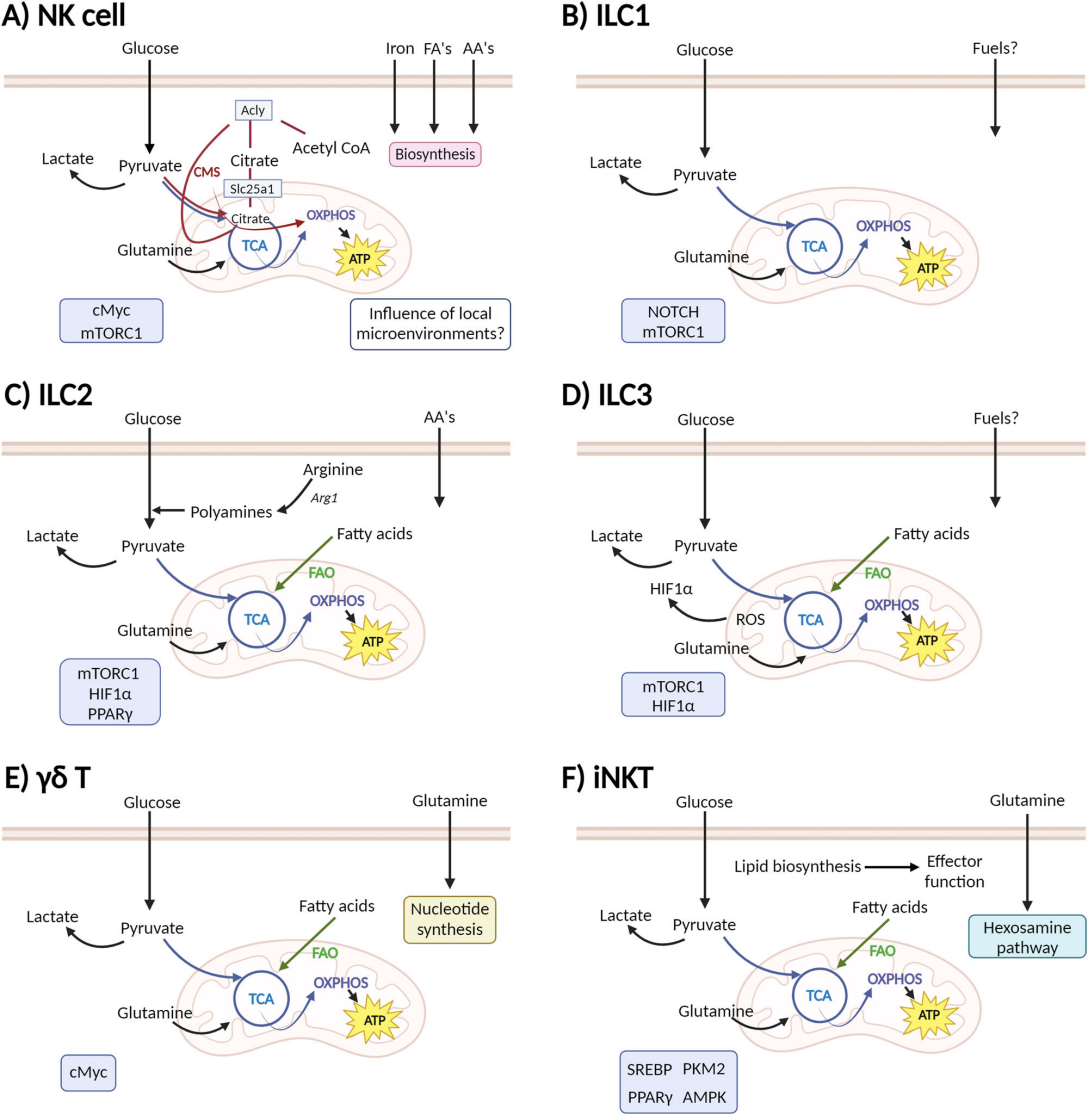
Overview of NK, ILC, γδ T and iNKT cell metabolism. Metabolic configurations described for innate lymphoid cells **A** NK cells, **B** ILC1s, **C** ILC2s and **D** ILC3. **E** γδ T cells present a metabolic dichotomy, with γδ^IFN^ relying on glycolytic metabolism, while γδ^17^ cells upon mitochondrial and lipid metabolism. **F** Metabolic configurations of iNKT cells are shown to be dependent on tissue location. Glycolytic metabolism is important for splenic and hepatic iNKT cells, whereas fatty acid oxidation is important for adipose tissue iNKT cells. Key signalling molecules identified in each cell type are shown in blue boxes. TCA tricarboxylic acid cycle (blue lines), OXPHOS oxidative phosphorylation, FAO fatty acid oxidation (green arrow), CMS citrate–malate shuttle (red lines). This figure created in BioRender. Finlay (2024) https://BioRender.com/c57f600.

Considering that innate lymphocytes inhabit diverse tissue niches, this begs the question whether the tissue microenvironment influences the metabolism of these cells. Also, are innate lymphocytes capable of tuning their metabolism to the different tissue environments they encounter? Much about what we know on the metabolism of innate lymphocytes comes from studies of cells in culture, and as such, this  data may not accurately reflect the metabolic configurations of the respective cells within the tissue niche. Regardless, innate lymphocytes at rest are metabolically inactive, relying on glycolysis-fuelled oxidative phosphorylation to support basal nutrient uptake and minimal biosynthesis. When activated, innate lymphocytes shift their metabolism to meet their energy needs, with increased metabolic rate and nutrient uptake to promote protein, lipid and nucleic acid synthesis. Metabolic configurations of these innate lymphocyte populations are outlined in Fig. 2. For the purpose of this review, murine studies will be primarily discussed unless stated otherwise. Additional considerations are required when studying other innate lymphocytes in humans such as mucosal-associated invariant T cells. Moreover, the emerging technologies discussed below for studying metabolic fluxes in single cells have largely been developed for mice, so additional challenges will be encountered when applying these techniques to human samples.

#### NK cell metabolism

NK cell metabolism in the context of health and disease has been reviewed extensively elsewhere^26,27^. NK cells exist in a relatively quiescent state, with basal metabolic rates that increase upon activation to support the production of cytokines and cytotoxic molecules. The signalling molecules, mTORC1, cMyc and SREBP, have all been implicated in the regulation of NK cell metabolic reprogramming^28–30^ (Fig. 2A). Deficiency of these signalling molecules, by either pharmacological or genetic approaches, results in decreased NK cell effector functions including reduced IFNγ production and killing capacity. Mice treated with a glycolytic inhibitor, 2-DG, or a mTORC1 inhibitor, rapamycin, show decreased NK cell IFNγ production following the injection of TLR agonist Poly(I:C), or the infection with mouse cytomegalovirus (MCMV)^30,31^. More recently, it was demonstrated that the uptake of iron and fatty acids (FA) is important to support NK cell metabolism and function in mice challenged with retrovirus infection^32,33^. Iron deficiency profoundly impairs NK cell antiviral functions, resulting in increased viral loads. These findings were connected to decreased expression of the transcription factor cMyc, decreased mitochondrial function, and reduced production of cytotoxic molecules^32^. Moreover, upon retroviral infection, splenic NK cells increase CD36 expression and FA uptake, while pharmacological inhibition of fatty acid β-oxidation impairs NK cell cytotoxicity^33^. A separate study showed that carnitine palmitoyltransferase I (CPT1)-dependent β-oxidation sustains NK cell mitochondrial function, actin polarisation and proliferation in MCMV infection and tumour models^34^. These studies highlight how NK cells can utilise multiple fuels to support their effector functions, and may thus have implications regarding how NK cells can alter their metabolism in environments with differing levels of iron and lipids. This is likely also true for other tissue-resident lymphocytes, yet their metabolism has not been studied to the same degree as NK cells.

#### ILC1 metabolism

It is assumed that ILC1s share comparable metabolic features to NK cells due to their similar phenotypic characteristics (Fig. 2B), but in actuality ILC1 metabolism is sparsely described, with just one study demonstrating that ILC1s are enriched for Notch and mTOR signalling pathways^35^, and another showing that following *Mycobacterium* infection lung ILC1-like cells are primarily glycolytic with low levels of mitochondrial metabolism^36^.

#### ILC2 metabolism

There have been more studies on ILC2 metabolism compared to that on ILC1 cells (Fig. 2C). ILC2s rely on non-glucose nutrients, including fatty acids and arginine, to fuel their metabolic pathways and support their effector function^8^. At rest, ILC2s are highly dependent on oxidative phosphorylation and have elevated mitochondrial polarisation and mass^37^. Indeed, mitochondrial metabolism is crucial for ILC2 homeostasis, as individuals with mitochondrial disease have severely reduced numbers of ILC2 cells^37^. Surace et al. demonstrated that human ILC2s have high expression of amino acid transporters, including LAT4 (Slc3a2/Slc43a2), which transports large neutral amino acids, and Slc1a5, which transports alanine and glutamine. Metabolomic analysis confirmed that ILC2s are enriched for amino acids such as branched-chain amino acids and glutamine^37^. Upon activation, ILC2s continue to utilise amino acids to fuel oxidative phosphorylation and support proliferation processes which are dependent on glycolysis and mTORC1 activity^37^. In the intestine, ILC2s use extracellular fatty acids to fuel β-oxidation. This metabolic feature has been studied during helminth infection where inhibition of fatty acid oxidation, but not inhibition of glycolysis, reduces ILC2 cytokine production^38^. In addition, upon chronic activation, ILC2s increase the uptake of glucose as well as external lipids stored as lipid droplets that are subsequently converted to phospholipids to sustain ILC2 proliferation. Feeding mice a ketogenic diet, thus reducing glucose availability, reduced ILC2 infiltration and cytokine production upon papain challenge^39^. In parallel, the expression of the enzyme arginase 1 (Arg1) is a distinguishing feature of ILC2 cells. Arg1 metabolises arginine to generate ornithine and urea, with further metabolic reactions generating proline and polyamines important for cell growth and proliferation. Expression of Arg1 is a conserved trait of ILC2s in both human and mouse lymphoid and non-lymphoid tissues^40^. Pharmacological inhibition of Arg1 enzymatic activity results in reduced levels of glycolysis and polyamine biosynthesis^40^. Deletion of Arg1 in murine ILCs restrains ILC2 proliferation and cytokine production within the lung^40^. While Arg1 is also expressed in a proportion of ILC3s, it is not critical for their development or function^40^.

#### ILC3 metabolism

ILC3s have been shown to take up glucose and fatty acids at rest, although the latter to a much less extent than ILC2s^38^. Upon ILC3 activation there is an enrichment in pathways associated with glycolysis, including pyruvate, fructose and mannose metabolism, based on single-cell transcriptome analysis^35^ (Fig. 2D). Supporting the role of glycolysis in ILC3s, defective mTORC1 signalling led to reduced number of ILC3s and decreased IL-17 and IL-22 production^8,41,42^. As ILC3s exist primarily within the gut, they are exposed to and influenced by metabolites from food intake and the inherent hypoxic gut environment. For instance, studies have shown that vitamin A deficiency results in reduced ILC3s within the gut, decreased ILC3 cytokine production, and increased susceptibility to bacterial infection^43^. Following *Citrobacter rodentium* (*C. rodentium*) infection, ILC3s increase cytokine production and proliferation as induced by mTORC1-HIF1α signalling, and show heightened glycolysis and production of mitochondrial ROS for stabilising HIF1α protein levels^42^. Hypoxia leads to increased ILC3 numbers, activation and proliferation, and mice lacking HIF1α in RORγt-expressing cells, which includes ILC3s, had reduced ILC3 cytokine production and increased susceptibility to *Clostridioides difficile* infection^41^. In addition, deletion of acetyl-CoA carboxylase 1, which converts acetyl-CoA to malonyl-CoA, in RORγt^+^ ILCs results in decreased IL-22 production, intestinal barrier dysfunction and increased susceptibility to *C. rodentium* infection^44^.

#### γδ T cell metabolism

The metabolism of γδ T cells is poorly described compared to that of αβ T cells (Fig. 2E). One study found that the fuel preferences and metabolic pathways engaged differed between γδ^IFN^ and γδ^17^ cells, with γδ^IFN^ cells relying primarily on glycolytic metabolism, while γδ^17^ cells preferentially utilising mitochondrial and lipid metabolism^45^. This metabolic dichotomy was established within the thymus during development, and was maintained when mature γδ T cells reached their tissue location. In the peripheral lymph nodes, γδ^17^ cells have significantly higher mitochondrial mass and membrane potential, while γδ^IFN^ cells have increased glycolysis-related genes and cMyc expression. In addition to their dependence on mitochondrial metabolism, γδ^17^ cells have increased lipid content and increased lipid uptake compared to γδ^IFN^ cells. Increased lipids from high fat diet or the lipid-rich B16 melanoma tumour microenvironment supported the expansion of γδ^17^ cells but not γδ^IFN^ cells^45^. In humans, this dichotomy has not been studied, but glutamine metabolism was shown to be essential for the production of IL-17A by activated γδ T cells^46^. These findings highlight that γδ T cells adopt distinct metabolic configurations that correspond to discrete effector functions.

#### iNKT cell metabolism

It has been shown that the metabolic regulators, mTORC1 and cMyc, are important for iNKT cell development and proliferation within the thymus, but persistent mTORC1 activation inhibits iNKT cell development^47^. Glucose and glutamine are both important fuels for iNKT cell development, homeostasis and function, with glucose supporting TCR recycling and IFNγ production in splenic and hepatic iNKT cells^48^, and with glutamine fuelling the TCA cycle via glutaminolysis to maintain redox balance and glycosylation processes through the hexosamine biosynthesis pathway^49^ (Fig. 2F). Lipid biosynthesis mediated by the transcriptional regulators, PPARy, Srebf1 and PLZF, is also an important process in iNKT cells^50^. Furthermore, depending on their locations, tissue-resident iNKT cells exhibit specific transcriptional programmes that adapt cellular metabolism to the local environment. For instance, splenic and hepatic iNKT cells have similar glycolytic metabolic profiles driven by increased PKM2 expression, with inhibition or deletion of PKM2 impairing the responses of iNKT cells in these tissues. By contrast, adipose-resident iNKT cells rely on AMPK signalling and use FA β-oxidation to support their function and maintain adipose tissue homeostasis^51^. Indeed, deletion of AMPK or inhibition of β-oxidation impairs adipose iNKT cell, but not splenic iNKT cells functional outputs^51^. These studies highlight the importance of investigating the metabolism of iNKT cells and immune cells within their tissue niche.

### Potential pitfalls when studying metabolism in cultured immune cells

Low cell number is one of the major limiting factors in analysing the metabolism of rare immune cell populations. While expansion of cells in culture is one approach to generate greater cell numbers, there is a recent appreciation that in vitro culture of immune cells can substantially alter their metabolism. Indeed, metabolic readings generated using cells cultured in vitro are very different to the metabolic features of that cell within the in vivo niche^52–54^. It is important to note that traditional cell culture mediums currently being used were formulated more than 50 years ago. These mediums were not developed to be physiologically relevant; instead, they were created to produce large amounts of cells with low variability. Hence, it is unsurprising that these mediums poorly represent metabolite concentrations in vivo. First established by Kaymak et al., Van Andel Institute-modified Iscove’s medium (VIM) has adjusted the concentrations of glucose and glutamine to better reflect physiological conditions. In addition, nutrients not normally found in traditional medium but present in murine serum are added to provide a more physiological method for culturing cells^55^. Nevertheless, even with the improvement made with VIM media, in vitro culture systems cannot fully recapitulate nutrient availability within the tissue, nor other parameters within the in vivo niche including oxygen levels and waste products.

The disparities between in vivo niches and in vitro culture conditions were first documented in tumour cells using in vivo infusion of ^13^C-labelled metabolites^52–54^. With this in mind, there has been a concerted effort to study the metabolism of immune cells in vivo using similar isotope labelling approaches. For instance, Ma et al. have compared CD8 T cells in vitro and in vivo following activation by the gram-positive bacteria *Listeria monocytogenes*^56,57^. While in vitro activated effector T cells were highly glycolytic, in vivo activated CD8 cells showed greater rates of oxidative metabolism, increased pyruvate entry into the TCA cycle as citrate, and the flow of ^13^C-glucose carbon was diverted to anabolic pathways such as serine and nucleotide biosynthesis^56^. Using ^13^C-labelled glutamine and acetate, it was shown that the metabolic dependencies of in vivo activated T cells change over time to support pyrimidine synthesis and ATP production, with acetate becoming a key fuel to support oxidative metabolism in T cells at later timepoints in the course of infection^57^. Together, these studies highlight that studying T cells metabolism within the in vivo niche is critical for truly understanding T cell function. It should be noted that several complementary methods, including proteomics and metabolomic approaches, were used in these studies in addition to stable isotope-tracing. Thus, a combination of various experimental techniques may be necessary to help create a comprehensive understanding of the metabolic needs of immune cells in vivo. However, the experimental setup for in vivo isotope-tracing experiments is complex, and this limits the scope of experimentation that can be performed and restricts these experimental approaches to specialised research groups.

### Advocating for single-cell metabolic analyses

While techniques are continually improving to better probe the metabolism of rare immune cell populations, our understanding of these processes is still lacking. As outlined above, this is partly due to cell number issues and the influence of non-physiological medium. Moreover, it is important to consider the tissue-specific niches where these cells reside, as nutrient and metabolite content can be altered by locations of immune cells relative to the tissue vasculature^53,58^, disease states such as diabetes or obesity, or physiological states such as fasting, feeding and exercise^59^. Therefore, it is key that new techniques are developed to accurately measure the metabolism of rare immune cell populations ex vivo immediately after tissue digestion, or ideally within their tissue niche to avoid any potential artifacts created during the digestion process.

### Single-cell analysis of metabolic features in rare immune cell populations

While technologies are currently available for, or are rapidly evolving towards, measuring metabolic features of immune cells with single-cell resolution, a number of challenges still exist due to the inaccessibility and intricacy of some single-cell methods. Thus, there is a need to develop approaches accessible-to-all to study metabolism in individual immune cells. Methodologies with such a potential are highlighted below.

#### Single-cell RNA sequencing

*Single-cell RNA Sequencing* (scRNA-seq) has revolutionised the study of rare immune cell populations by identifying gene expression profiles at the single-cell level (see ref. ^60^ for scRNA-seq best practices and guidelines). However, scRNA-seq has significant limitations as a technique to study the metabolism of single cells. While scRNA-seq can unveil a considerable amount of transcriptional information, there is often a poor correlation between transcriptomes and proteomes^61,62^. mRNA levels can provide insights into a cell’s metabolic configuration but do not accurately inform cellular metabolism, as proteins and enzymes are responsible for driving the metabolic flux, yet mRNA levels do not reliably reflect protein levels or enzymatic activity^61,62^.

Despite the caveats of scRNA-seq as a method to study the metabolism of single immune cells, this technique has provided some of the first datasets with insights into the metabolism of NK cells in human tissues. Netskar et al. generated a reference map of healthy blood- and tissue-derived NK cells and then incorporated data from publicly available datasets to state the differences induced by the tumour microenvironment of seven solid tumours. Interrogating these mRNA datasets revealed some interesting differences in the metabolic features of NK cells in different types of tumours that would be interesting to confirm using additional metabolic analyses^63^.

That said, advances in scRNA-seq technologies are increasing the value of scRNA-seq as a method to survey the metabolic configurations of tissue-resident immune cells. A modification of scRNA-seq, CITE-seq, uses oligonucleotide barcode-tagged antibodies to label surface epitopes on cells for quantification of protein levels^64^. Initially used to provide greater confidence in immune cell subset identification, CITE-seq could be adapted to quantify the expression of metabolic proteins, such as nutrient transporters, on the cell surface thus allowing the correlation of cellular transcriptomes with the protein expression of key metabolic machinery. However, one caveat of CITE-seq is that quantifying intracellular metabolic proteins and enzymes remains challenging as cell fixation is required, causing technical difficulties for RNA sequencing.

**Fig. 3:**
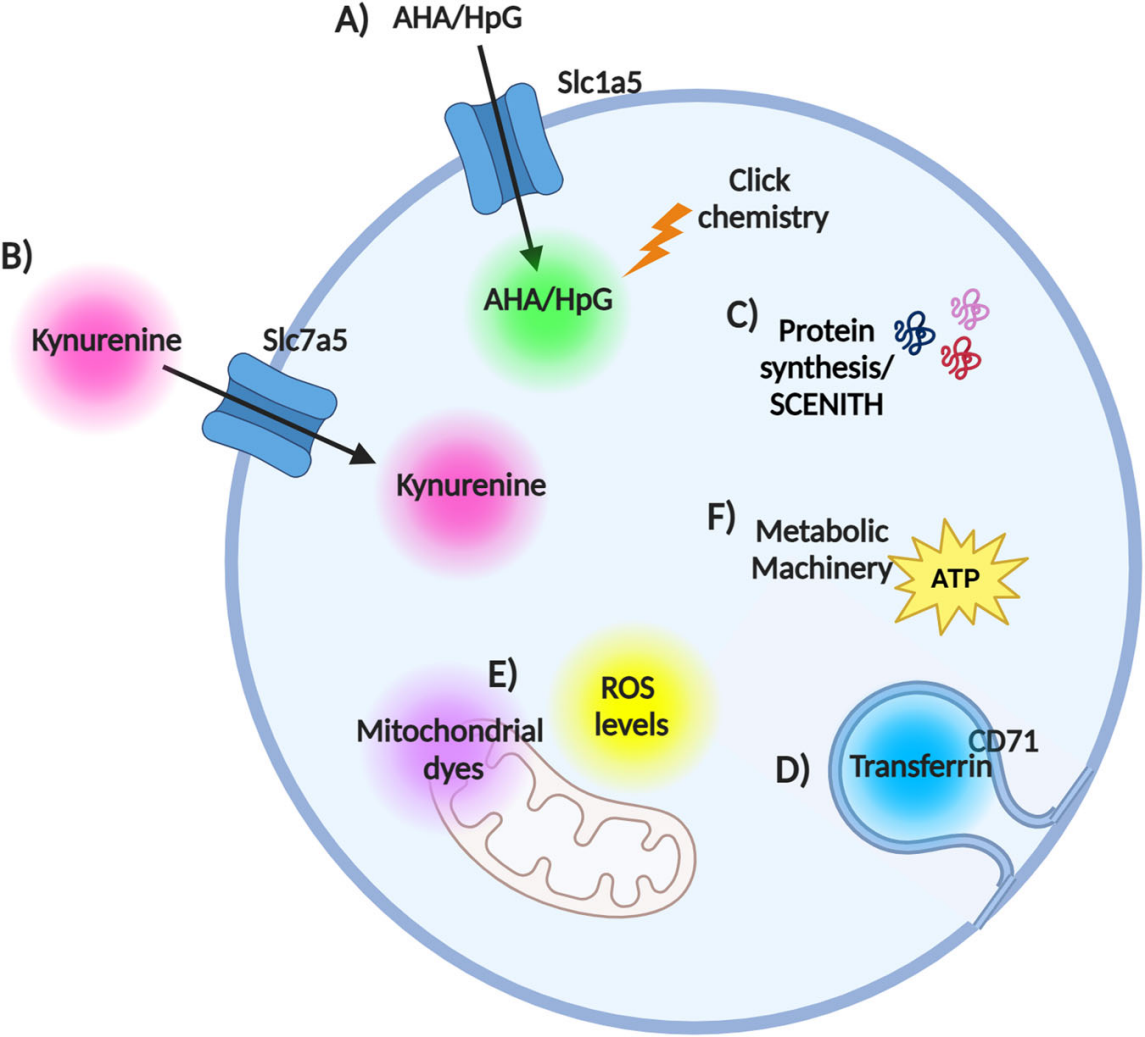
Overview of current flow cytometry techniques available to probe cellular metabolism. **A** Glutamine (Q) uptake assay with single-cell resolution (QUAS-R); bioorthogonal amino acids l-azidohomoalanine (AHA) and l-homopropargylglycine (HPG) are used to quantify amino acid transport through Slc1a5. **B** Kynurenine is a naturally fluorescence cargo for Large neutral amino acid (LAT1-4) transporters and is used to measure amino acid uptake through these transporters. In many lymphocytes, LAT1 (Slc7a5/Slc3a2 heterodimer) is the only LAT isoform expressed and so kynurenine uptake quantifies Slc7a5 mediated amino acid uptake. **C** SCENITH estimates single-cell energetic metabolism by profiling translation inhibition. Puromycin incorporation is used to quantify protein translation rates and to probe energy metabolism. **D** Iron–transferrin uptake is measured using a fluorescently tagged transferrin molecule. **E** Mitochondrial dyes can be used to measure mitochondrial mass, mitochondrial ROS and polarisation across the mitochondrial inner membrane. **F** METFLOW is a high-parameter flow cytometry method utilising antibodies against metabolic proteins; that probes the capacity of a cell to engage different metabolic pathways. Essential to the accurate interpretation of these assays are robust controls; for instance, competition controls are imperative for QUAS-R and Slc7a5 uptake analysis. Protocol A is compatible with protocol B, and protocol C. Further effort is being made to integrate the remaining parameters. This figure was created in BioRender. Finlay (2024) https://BioRender.com/d06l575.

**Fig. 4:**
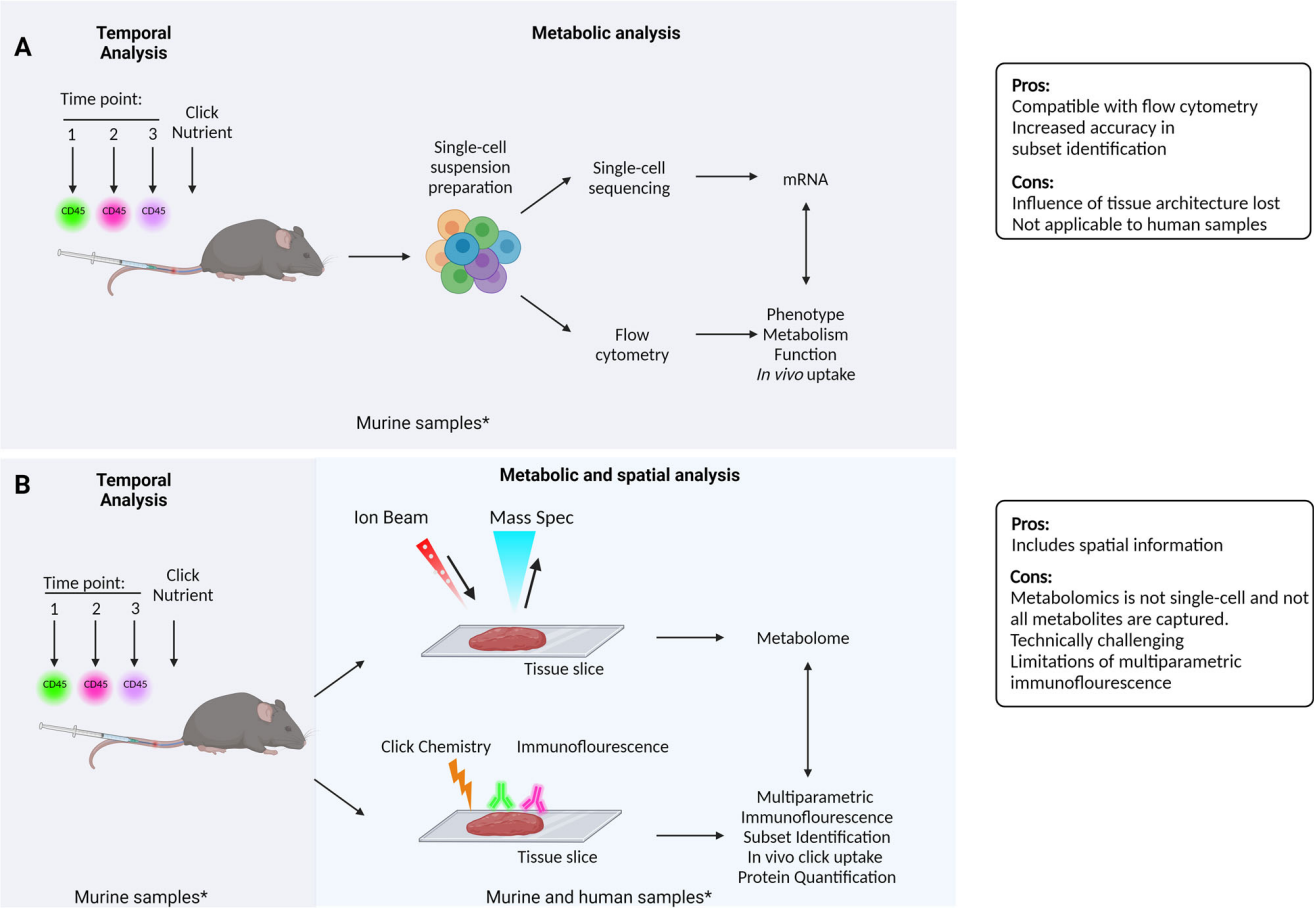
Potential mechanisms for integration of techniques discussed. **A** Temporal analysis of immunometabolism. In mice, time-stamping and in vivo nutrient uptake might be combined to gain understanding of how the metabolism of tissue-resident innate lymphocytes changes with the length of time they are within a tissue. With this format, flow cytometry and scRNA-seq platforms can be integrated to gain insight into the transcriptome and metabolic features, including in vivo uptake, of immune cells of interest and correlate this with cellular location at the time of sample retrieval. While spatial information is mostly lost in this approach, the in vivo uptake data will reflect whether a given cell had access to the click-nutrient and this will be impacted by the tissue architecture and competition for the click-nutrient between cells. **B** Spatial and temporal analysis of immunometabolism. This approach relies on the use of tissue sections where adjacent sections are processed for spatial metabolomics (top) or multiparametric immunofluorescence (bottom). In human samples this approach might be used to generate information about the metabolome of the tissue section and how spatial metabolite levels relate to the location of innate lymphocytes, identified using immunofluorescence in an adjacent tissue section. With advances in multiparametric immunofluorescence, metabolic features of innate lymphocytes could be measured using antibodies specific to metabolic machinery (nutrient transporters, enzymes) or metabolic signalling molecules (such as mTORC1 signalling components). In mice, the time-stamping and in vivo nutrient uptake approaches described in (**A**) could be added to the protocol, though this would add additional complexity to the multiparametric immunofluorescence. There are significant challenges involved in optimising such an experimental setup. This figure was created in BioRender. Finlay (2024) https://BioRender.com/b20j926.

Additional advances are unlocking ability to analyse the transcriptome of immune cells in space and time. Until recently, scRNA-seq was limited to capturing static gene expression and lacked temporal aspects. A study from Kirschenbaum et al. defined a new technology for scRNA-seq, coined “Zman-seq”, which utilised in vivo labelling methods (“time-stamping”) to resolve scRNA-seq data for NK cells based on the duration that they were exposed to the tumour microenvironment^65^. These “time-stamping” techniques can be further integrated to other flow cytometry-based techniques as outlined in Figs. 3 and 4. A recent study utilised spatial CITE-seq to profile more than 180 proteins and associated single-cell transcriptomes in mouse (spleen, intestine and kidney) and human (spleen, tonsil, thymus and skin) tissue slides^66^. Taken together, quantifying metabolic proteins, determining the time spent within a given tissue, and understanding the spatial location of an immune cell with a tissue slice should all significantly increase the utility and versatility of scRNA-seq for studying the metabolism of tissue-resident innate lymphocytes.

#### Towards single-cell proteomics

Understanding protein expression data is a step closer to understanding metabolic processes within immune cells. High-resolution quantitative proteomics has been used to provide insights into the metabolism of immune cells in numerous studies, and has also been used to measure the copy numbers for greater than 7000 proteins^62,67–69^. Through this technique, it was demonstrated that cMyc is a key regulator in murine T cell function, controlling T cell activation and metabolic reprogramming^67^. Moreover, this technique was also used to describe the effect of gut microenvironment on tissue-resident intestinal intraepithelial T lymphocytes (T-IEL). Here, it was demonstrated that T-IELs have increased expression of cell surface receptors involved in epithelial interactions and upregulated cholesterol and lipid metabolic pathways in comparison to CD8 T cells isolated from lymph nodes^69^. In these studies ~10^6^ cells were used per sample, numbers that cannot reasonably be isolated for most tissue-resident innate lymphocytes.

Only recently are technological advances allowing the identification and quantification of proteins by mass spectrometry in a single cell. Bennett et al. intensely reviewed the state of art for these techniques, including the challenges faced, the technological advancements, and future development for the field^70^. Initial studies took advantage of larger cells with abundant protein content, such as neurons and oocytes^71–73^, or cell lines^74,75^. However, immune cells, particularly lymphocytes, present a particular challenge as they are small cells with low protein content^76^. The work of Petelski et al. described the Single Cell Proteomic (SCoPE2) protocol in which cells are isolated by sorting into multiwell plates and require minimal sample preparation, an approach that might be applied to investigate tissue-resident cell metabolism as the sensitivity of mass spectrometers advance^74^. Additionally, the platform described by Keren et al., called MIBI-TOF, integrates a mass spectrometer for multiplexed ion beam imaging with orthogonal acceleration time-of-flight. With that, they could profile the subcellular expression and localisation of 36 elementally labelled antibodies to probe phenotypical and metabolic proteins (including HIF1α, IDO-1 and phosphorylated S6 ribosomal protein), as well as endogenous elements, such as iron, in human tissue biopsies^77^. At present, while proteomics cannot deliver on single-cell proteomes for tissue-resident innate lymphocytes, recent technology advances could provide high-resolution quantitative data for bulk-sorted immune cells using very low cell numbers such as 10^2^–10^3^ cells, which is within range of what can be obtained for innate lymphocytes populations from a single tissue (personal communication). This approach would allow for a robust analysis of the metabolic proteome of tissue-resident innate lymphocytes but would not capture the metabolic heterogeneity of these cells within a tissue.

#### Towards single-cell metabolomics

Compared to mRNA and protein abundance, quantifying metabolites levels is a more accurate indicator of the metabolic processes in a cell. High-sensitivity bulk metabolomics has proven to be a crucial technique in exploring immune cell metabolism, providing insights into alterations in metabolite levels and metabolic pathways. Through this technology, the researcher can identify metabolic signatures of immune cells both basally and in various disease states. For example, Lozada et al. revealed differences in the metabolic features of human NK cells from individuals previously infected with Cytomegalovirus (CMV), demonstrating increased intermediates of the pentose phosphate pathway^30^. Using stable isotopic labelling methods, it is possible to trace the flux of different atoms through metabolic pathways in immune cell populations. For example, using ^13^C labelled glucose, Assmann et al. demonstrated that cytokine-activated murine NK cells use a glucose-fuelled non-canonical TCA cycle called the citrate–malate shuttle to support mitochondrial energy production^28^. It is also important to consider that standard bulk metabolomics analysis requires pure cell populations typically achieved by procedures including magnetic bead sorting or fluorescence-activated cell sorting (FACS), but this is complicated by the fact that sheath fluid from a FACS sorting can significantly impact the cellular metabolome due to the depletion of cellular metabolites^78^. In addition, these approaches typically require a large number of cells, making adopting these approaches challenging, if not impossible, on rare immune populations such as tissue-resident innate lymphocytes.

Methodologies are rapidly advancing to meet the challenges of measuring the wide range of metabolite species in low sample volumes and even in single cells^79^. The work of Schönberger et al. described a workflow to analyse intracellular metabolites from a small number of cells by FACS-sorting them directly into the extraction buffer. They could detect six metabolites above the background level in as little as 100 B cells and up to 80 metabolites in 5000 cells^80^. Indeed, single-cell metabolomic platforms would be a powerful tool that would not only unlock the metabolism of tissue-resident innate lymphocytes but also reveal the metabolic heterogeneity within these immune cells populations. However, a number of challenges remain before the promise of single-cell metabolomics can be fully realised including the misidentification and annotation of metabolites. Many metabolites lack reference standards and there is an aspect of uncertainty in metabolite identification, especially in untargeted metabolomics studies, where 10s–1000s of metabolites are considered of biological significance and need to be identified^81^, thus making the correct identification and quantification difficult. A recent comment published in *Nature Metabolism* highlighted some of these issues^81^.

Mass spectrometry imaging using technologies including matrix-assisted laser desorption/ionisation (MALDI), desorption electrospray ionisation (DESI), and secondary ion mass spectrometry (SIMS) has the potential to be a powerful tool in studying lymphocytes within their tissue niche^82^. Mass spectrometry imaging can be applied to derive metabolite information with 10–100 μm resolution. Rappez et al. report a method called SpaceM that integrates MALDI-based metabolomics and fluorescence imaging to identify >100 different metabolites in individual cells^83^. Another method developed by Yuan et al. integrated metabolite features generated by time-of-flight secondary ion mass spectrometry (TOF-SIMS) and H&E staining with computational methods to explore the spatial metabolome and tissue anatomy at single-cell level^84^. However, this approach cannot identify the specific tissue-resident immune cells within a given tissue. Ganesh et al. reported a method that used an isotope-tagged antibody library to achieve cell type-specific, three-dimensional spatially resolved metabolomic profiling framework (3D-SMF), with which they identified metabolite features of immune cells in human tonsil samples^85^. Recently, Hu et al. reported the Single-Cell SPAtially resolved METabolic (scSpaMet) framework that combines 3D-SMF with multiplexing proteomic imaging mass spectrometry to generate a single-cell map with >200 metabolites and 25 protein markers per cell, and they showed cell type-specific local metabolic competition in lung cancer tissues and human tonsils^86^.

Regardless of these advances, challenges still remain for spatial mass spectrometry imaging metabolomics to generate more detailed metabolic maps with greater sensitivity and depth of coverage of diverse cellular metabolites while accurately resolving for individual immune cells within spatially crowded tissues sections. Therefore, the potential of these technologies to revolve the immunometabolism of rare tissue-resident immune cells has not yet been realised.

#### Flow cytometry-based metabolic analyses

Flow cytometry is arguably the most valuable tool for studying cellular metabolism of rare tissue-resident population for a number of reasons. First, flow cytometry is the preferred method used by immunologists, so flow cytometry-based approaches are essentially accessible to all researchers studying immunometabolism. Second, by allowing multiple parameters to be analysed simultaneously, including functional and phenotypical features, flow cytometry is the workhorse of those studying rare immune cell populations. Additionally, flow cytometry platforms have the capacity to provide information for immune cells ex vivo with minimal cell manipulation or processing. Advanced flow technologies including time-of-flight mass spectrometry and heavy metal-labelled antibodies (mass cytometry) and fluorescent spectral flow cytometry analysers now allow for detection of over 40 parameters^87^. While with less throughput, imaging flow cytometry can now provide single-cell information with subcellular spatial information. Below we describe the flow cytometry-based assays of single-cell metabolic flux that can be applied to rare immune subsets, something that cannot be achieved by scRNA-seq, proteomics or metabolomics techniques.

##### SCENITH

A method developed by Argüello et al.^88^, SCENITH adapts the fact that nearly half of a mammalian cells energy, in the form of ATP, is consumed by the process of protein translation^89–91^. Measuring rates of translation can thus serve as a proxy for the abundance of ATP in the cell. Puromycin is incorporated into nascent polypeptides in a way that correlates to the rates of translation. The abundance of puromycin in nascent polypeptides can then be quantified using a fluorescent anti-puromycin antibody and flow cytometric analysis. Additionally, SCENITH probes which metabolic pathways, such as glycolysis or OXPHOS, contribute to ATP production in each individual cell. In this regard, a given biological sample is divided and treated with or without metabolic inhibitors that target glycolysis (2-deoxyglucose), or mitochondrial ATP synthesis (oligomycin). The effects of individual metabolic inhibitor, or set of inhibitors, on puromycin incorporation then allows the researchers to infer which metabolic pathways are used by each cell to produce ATP. For example, Corral et al. employed SCENITH to demonstrate that the differentiation of an ILC precursor to ILC1-like cells in the lung is associated with the metabolic reprogramming towards glycolysis^36^. Importantly, this technique is compatible with staining for intracellular markers that are essential for the correct characterisation of some subsets of tissue-resident lymphocytes. Increasing number of studies have utilised this technique, though there is a lot to be explored^45,92–94^. For example, SCENITH could be adapted to use low-dose of etomoxir, an inhibitor of CPT1A to probe β-oxidation in a given cell, as showed by Schimmer et al. for NK cells^33^. Alternatively, an inhibitor of ACLY, such as SB204990, could be used to investigate how the citrate–malate shuttle (a non-canonical TCA cycle) contributes to ATP production. Lastly, click chemistry-compatible analogues of puromycin, such as o-propargyl-puromycin, can also be used for the SCENITH method and have potential for reducing non-specific signals on account of the highly specific nature of bioorthogonal chemical reactions^95^.

##### Nutrient uptake assays

Click chemistry is a framework for performing highly specific chemical reactions between two chemical groups, not found in nature, within the complex chemical environment of the cell. The work leading the development of this technology was awarded with the Nobel Prize in Chemistry in 2022. Click chemistry has recently been utilised to study single-cell nutrient uptake by immune cells^96^. The work of Pelgrom et al. demonstrated that two bioorthogonal amino acids are transported by the glutamine transporter Slc1a5. These amino acid analogues have a small modification that introduces a functional chemical group that can react with other non-native compounds by click chemistry. Fluorophores that contain the click-reactive group are commercially available and the reaction will snap the amino acid and fluorophore together. Attaching the fluorophore after the glutamine analogue has been transported into the cells provides a fluorescent signal that is quantified by flow cytometry to give Slc1a5 activity in each individual cell. The study demonstrated the applicability of the assay both ex vivo and in vivo, which makes it a valuable technique in the analysis of tissue-resident cells. Slc1a5 is the predominant glutamine transporter in many immune cells^97^ and analysing its role in different contexts such as tissues and diseases will be pivotal in future studies.

Similarly, the activity of another amino acid transporter Slc7a5, responsible for transporting large neutral amino acids into cells, can also be probed by flow cytometry. In 2018, Sinclair et al. demonstrated that the fluorescent properties of kynurenine could be employed to quantitatively measure the activity of Slc7a5 in T cells^98^. Slc7a5 is an important transporter in many immune cells through providing amino acids into the cell but also for supporting anabolic signal transduction through mTORC1 and cMyc^29^. This kynurenine-based uptake assay has been employed to quantify Slc7a5 activity in a wide array of immune cells and immune contexts.

Finally, these uptake assays are not limited to amino acids either. Iron exists in the body bound to transferrin and the uptake of iron-bounded transferrin is mediated by the CD71 receptor^99^. Through the use of a fluorescent transferrin conjugate, endocytosis through CD71 can be investigated. This technique was used to demonstrate the importance of iron uptake into NK cells during acute retroviral responses^32^.

##### Metabolic machinery

Another flow cytometry-based approach to analyse the metabolism of immune cells is to quantify the metabolic machinery. Both Ahl et al.^100^ and Hartmann et al.^101^ described methods that combine a range of antibodies against metabolite transporters, metabolic enzymes and signalling molecules to probe human immune cell metabolism^101^. These approaches used standard or mass cytometry, respectively, and showed dynamic metabolic states across immune cell populations. Others have then successfully applied the techniques for mouse studies^102,103^.

##### Mitochondria

A core component of the metabolic machinery within a cell are the mitochondria, and mitochondrial dynamics are crucial for sustaining immune cell function. A number of fluorescent mitochondrial dyes and transgenic mice make it possible to probe mitochondrial fitness by flow cytometry. Fluorescent mitochondrial dyes such as MitoTracker Green and tetramethylrhodamine methyl ester measure mitochondrial mass and membrane potential, respectively^104^. 10-*N*-nonyl acridine orange is another probe for mitochondrial mass because it has high affinity for cardiolipin, specific to the mitochondrial membrane^105^, while levels of mitochondrial reactive oxygen species can be measured using MitoSOX and similar dyes. Used in combination, these dyes can provide an overview of mitochondrial health within cells^104,106^. However, it should be noted that these parameters should be used with caution, as some off-target effects and toxicity have been reported for these mitochondrial dyes^107^. It is recommended to use appropriate controls and the lowest concentration possible when analysing these parameters.

Aside from these mitochondrial probes, a number of specialised transgenic mice have been generated to investigate mitochondrial health as well as the activity of mitophagy, the process of autophagic turnover of mitochondria. Mitophagy reporter mice express a mitochondrial-targeted pH sensitive fluorescent probe that changes its fluorescent output when the mito-autophagosome fuses with the lysosome at a late stage of mitophagy^108^. The PhAM^floxed^ mouse line facilitates the expression of a mitochondrially localised version of Dendra-2, a photo-convertible fluorescent protein^109^. This allows a more consistent probing of mitochondrial mass, compared to mitochondrial dyes, and has been applied by Aguiar et al. to study mitochondrial morphology in rare tissue-resident immune cells such as iNKT cells^51^. The photo-conversion properties of Dendra-2 also allow analysing the process of mitochondrial fusion. Lastly, Russo et al. described a transgenic mouse method called Single-cell Profiling and Imaging of Cell Energy Metabolism (SPICE-Met), which measures the intracellular ATP:ADP ratio through the expression of a fluorescent sensor^110^.

##### Cell–cell interactions

To date, studies on immunometabolism lack information on the impact of cell–cell interactions on metabolic pathways. An exciting new technique, called universal labelling immune partnerships by SorTagging intercellular contacts (uLIPSTIC), records cell–cell interactions based on the enzymatic transfer of a biotin-labelled substrate that can later be detected by flow cytometry^111^. The integration of this method with other flow-based metabolic assays could expand our knowledge on the importance of physical interactions between immune cell types for modulating the metabolic programming of cells.

In summary, the integration of the techniques discussed above can provide multiple dimensions of metabolic flux analysis in individual cells, especially those found in low frequency in tissues (Figs. 3 and 4). The association of temporal and metabolic analysis, for example, could be made by taking advantage of injecting labelled antibodies in mice at different timepoints and later analysing the metabolism of immune cells by flow cytometry-based techniques, such as SCENITH, nutrient uptake and/or mitochondrial probes. This would provide valuable information in different disease models but also on how immune cells adapt their metabolism to different tissues under homeostatic conditions. In this regard, the integration of spatial and metabolic analysis has already been demonstrated^77^, but still faces challenges on the range of metabolites that can be analysed, and may require adaptations to integrate some of the nutrient uptake analyses to be detected in tissue slides. Thus, flow cytometry-based metabolic analysis have the potential to generate a multidimensional ex vivo metabolic footprint of individual immune cells and reveal new insights into the immune heterogeneity within and between tissues, in homeostasis and disease states.

### Conclusions and future perspectives

While studying tissue-resident immune cell metabolism is now achievable, there are several challenges remaining. Glucose metabolism is important for most immune cells, but there are no accurate flow cytometry-based techniques to define changes in glucose uptake and use within immune cells. This challenge is compounded by the continued erroneous use of fluorescent glucose analogue, 2-NBDG, as a measure of glucose uptake. Sinclair et al. provide conclusive proof that 2-NBDG is not transported by the glucose transporters expressed by the majority of immune cells, Slc2a1 and Slc2a3^112^, likely due to the attachment of large fluorophores changing the physical and chemical properties of a nutrient and unsurprisingly the transport characteristics of that nutrient. Similar artifacts are true for BODIPY-associated lipids that are commonly used to analyse lipid uptake. A recent study, however, provides the promise of measuring glucose uptake by flow cytometry using a bioorthogonal glucose probe, though this approach still requires careful testing in immune cells^113^.

Species-specific differences on the study of immunometabolism present several challenges due to variations in physiology, genetics, developmental timing and immune system function^114^. There are notable differences in metabolic pathways and immune responses that can impact the relevance of findings in mice to human health. For instance, certain immune cell populations, such as macrophages, behave differently in mice compared to humans^1^, leading to divergent outcomes in disease studies. Additionally, the metabolic environments in which immune cells function can vary significantly between the two species, thereby affecting how immune responses are regulated. Some of the techniques described here, such as the Zman-seq, involves injecting anti-CD45 antibodies at different timepoints, and therefore cannot be directly translated to human studies. All together, these differences necessitate careful consideration when extrapolating mouse data to human conditions, or vice versa.

**Fig. 5:**
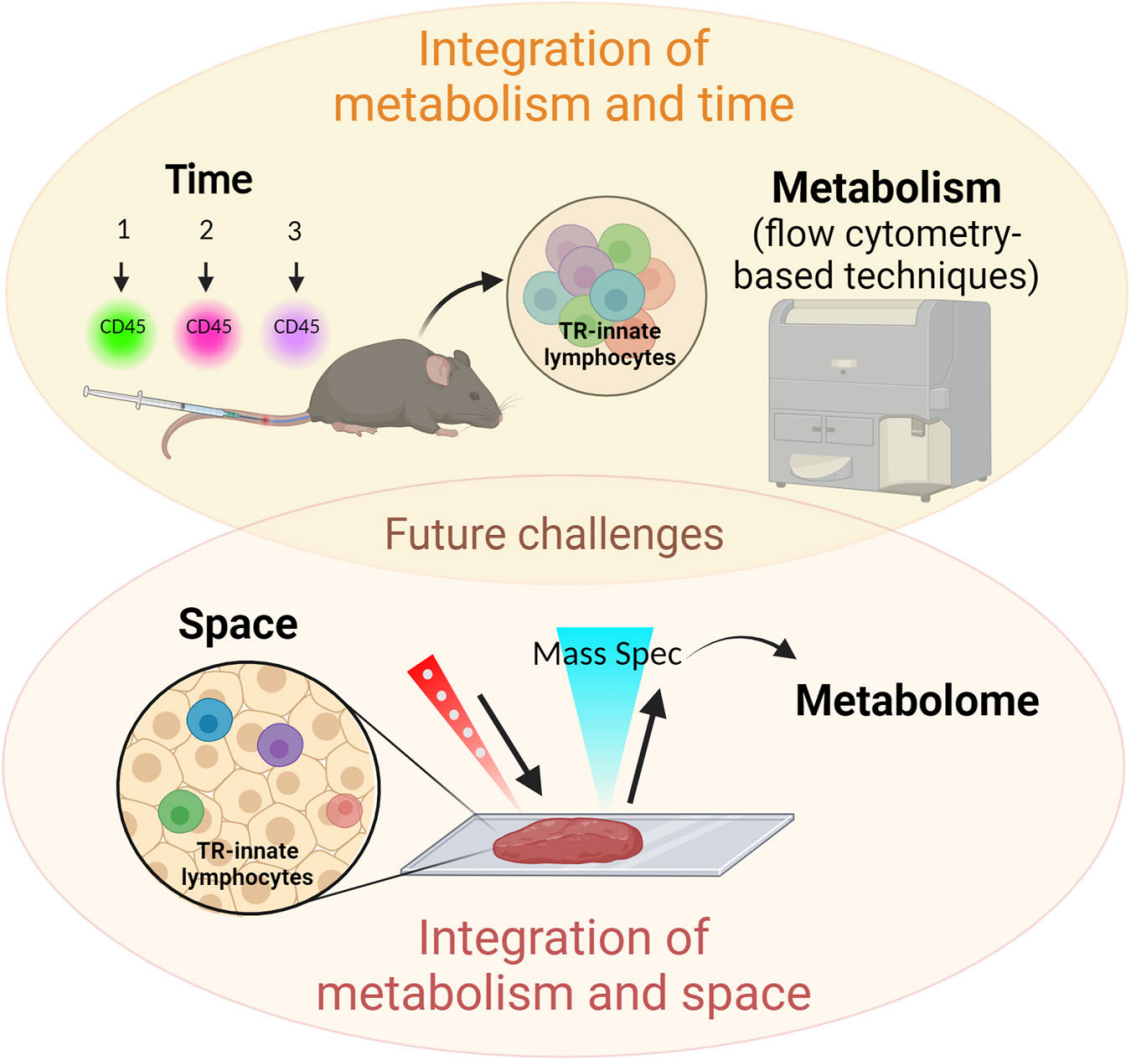
Integration of single-cell metabolic assays. Measuring immune cell metabolism with single-cell resolution will be crucial in unlocking the metabolic features of rare tissue-resident lymphocytes. Achieving this while retaining information about space, the tissue location of the cell, and probing the time spent in a given tissue will be another step towards understanding the true metabolism of these cells in situ within tissues. This figure was created in BioRender. Finlay (2024) https://BioRender.com/q29i709.

In summary, tissue-resident immune cells play an important role in maintaining tissue homeostasis and repair or influencing the development of diseases. The metabolic reprogramming of these cells is tightly connected to their function, but accurately measuring the metabolic parameters of these rare subsets remains extremely challenging. Therefore, it is imperative that we develop new strategies to explore the metabolic profile of these immune cells. In this review, we provided a comprehensive examination of current methodologies employed for the analysis of rare immune cell populations. Ideally, in the future, we will be able to integrate functional, metabolic, spatial and temporal data in the analysis of tissue-resident immune cells and advance the knowledge to develop strategies to manipulate these cells as a therapeutic approach (Fig. 5).
